# Relationship between Preoperative Nutritional Status and Clinical Outcomes in Patients with Head and Neck Cancer

**DOI:** 10.3390/nu14245331

**Published:** 2022-12-15

**Authors:** En-Ying Wang, Mu-Kuan Chen, Ming-Yu Hsieh, Chew-Teng Kor, Yen-Tze Liu

**Affiliations:** 1 Department of Otorhinolaryngology, Head and Neck Surgery, Changhua Christian Hospital, Changhua 500, Taiwan; 2 Department of Post-Baccalaureate Medicine, College of Medicine, National Chung Hsing University, Taichung 402, Taiwan; 3 Big Data Center, Changhua Christian Hospital, Changhua 500, Taiwan; 4 Graduate Institute of Statistics and Information Science, National Changhua University of Education, Changhua 500, Taiwan; 5 Department of Family Medicine, Changhua Christian Hospital, Changhua 500, Taiwan; 6 Oral Cancer Research Center, Changhua Christian Hospital, Changhua 500, Taiwan

**Keywords:** head and neck cancer, prognostic nutritional index, postoperative complication, survival

## Abstract

The nutritional status in cancer patients is related to cancer survival and surgical outcome. The objective of this study was to examine the relationship between preoperative prognostic nutritional index (PNI) and post-operative clinical outcomes in head and neck cancer (HNC) patients. A total of 1282 head and neck cancer patients receiving surgical resection in Changhua Christian Hospital between 1 January 2010 and 30 August 2021 were recruited in the final analysis after undergoing propensity score matching analysis. The logistic regression model was used to assess the association of the PNI group with overall and various complications. The patients in the high PNI group had a significant lower incidence of overall complications, medical complications, and pulmonary complications; but not significant surgical complications. The high PNI group had lower mortality risk. The results in this study revealed that PNI score was a significant independent predictor of postoperative complications in HNC patients undergoing surgical resection. We recommend preoperative testing and evaluation of HNC patients to identify low PNI and high-risk groups for postoperative surveillance.

## 1. Introduction

Head and neck cancers (HNC) consist of several malignancies, such as cancers of the oral cavity, nasal cavity, oropharynx, nasopharynx, hypopharynx, larynx, and salivary glands. According to the data published by the International Agency for Research on Cancer (IARC), more than 900,000 newly-diagnosed cases of HNC and 460,000 deaths occur each year [1]. Except for nasopharyngeal cancer, which is sensitive to chemo-radiotherapy, surgical resection with adjuvant therapy in high risk patients and radiotherapy remained as the two main treatment strategies of HNC.

The nutritional status in cancer patients is related to cancer survival, cancer progression, and surgical outcome [2,3,4]. Malnutrition leads to an increased rate of postoperative complications. The prognostic nutritional index (PNI) calculated by serum albumin level and lymphocyte count is a simple method to describe the nutritional status of patients with inflammatory diseases and malignancies [5,6,7]. However, the relationship between preoperative PNI score and postoperative outcomes has not been well-established in HNC patients receiving surgical resection. The objective of our study was to compare the clinical outcomes in different levels of preoperative PNI scores in patients with HNC receiving resection operations.

## 2. Materials and Methods

### 2.1. Study Populations

This study was a retrospective, observational cohort study conducted at Changhua Christian Hospital, (CCH) which is a tertiary medical center in Taiwan. The Changhua Christian Hospital Clinical Research Database (CCHRD) is an integration of all electronic medical record systems, which includes the cancer registry database, hospitalization records, prescriptions, laboratory data, clinical visit records, and death records at CCH. We included a total of 3415 patients who received HNC resection surgery at CCH between 1 January 2010 and 30 August 2021, and the patients’ data in CCHRD were screened.

**Figure 1:**
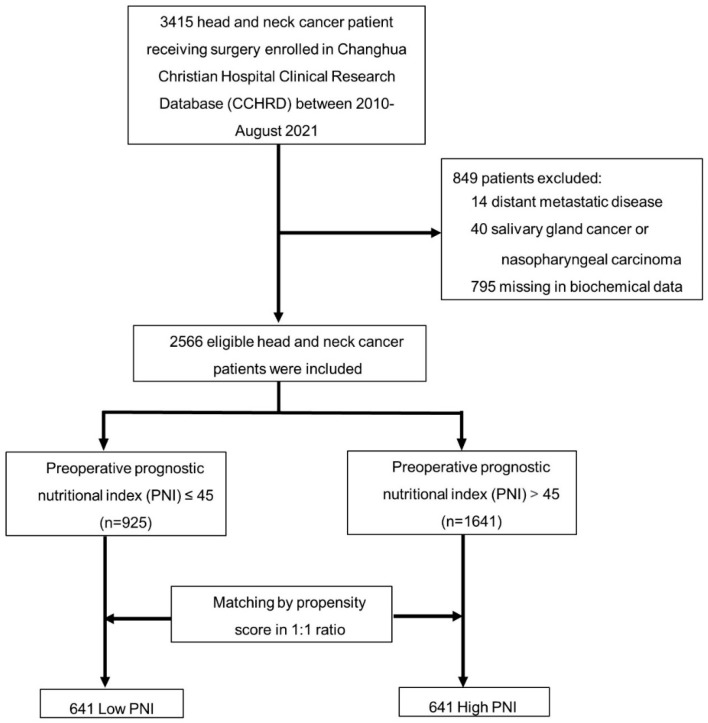
Study Flow chart. A total of 2566 out of 3415 patients were included in this study. Patients were categorized according to PNI score. A multivariate logistic regression analysis and propensity score-matched analysis were performed for the two groups. (PNI = prognostic nutritional index).

To identify how preoperative nutritional status had an impact on the clinical outcome in HNC patients, our study excluded patients with distant metastases, patients diagnosed with salivary gland cancer or nasopharyngeal carcinoma, and patients with incomplete biochemical data. We enrolled a total of 2566 HNC cases in our study (Figure 1). Our study was approved by the Institutional Review Board of CCH (IRB No: 211015).

### 2.2. Definition of Prognostic Nutritional Index and other Confounders

The preoperative PNI for each patient was calculated by the formula shown below, and patients were divided into low and high PNI groups based on established cut-off values: low PNI (<45) and high PNI (≥45) [8,9,10].

$\mathrm{PNI}\;\mathrm{score}=10\times \mathrm{serum}\;\mathrm{albumin}\left(g/\mathrm{dL}\right)+0.005\times total\;lymphocyte\;count\left(/\mathrm{mm}3\right)$ [11].

Patients’ demographics and clinical characteristics were obtained, including age, sex, education status, tumor-node-metastasis (TNM) stage of HNC, tumor grade, primary site of cancer, smoking, betel nut chewing and alcohol consumption history, Eastern Cooperative Oncology Group (ECOG) score, smoking and alcohol history, operating time, preoperative laboratory profiles (white blood cell count (WBC), hemoglobin (Hb), platelet count (PLT), red blood cell distribution width (RDW), absolute lymphocyte count, neutrophil, albumin, Alanine Aminotransferase (ALT), and creatinine, comorbidities (hypertension, diabetes mellitus, coronary artery disease, chronic kidney disease, chronic lung disease), and treatment modalities received. We collected all confounding data from CCHRD.

### 2.3. Endpoint

The primary endpoint of our study was defined as complications which occurred within 30 days after surgery. The complications recorded in our study were categorized into medical complications, pulmonary complications, and surgical complications. Medical complications discussed in this study were acute myocardial infarction, stroke, any condition needing resuscitation, or any condition that led to death. Pulmonary complications were defined according to postoperative chest X-ray findings and body temperature >38 degrees C with a positive result of sputum culture. Surgical complications were defined as wound dehiscence, wound infection, poor wound healing, or postoperative wound bleeding requiring re-operation.

The secondary endpoint of our study was to examine whether the preoperative PNI had a long-term effect on the recurrence and survival rates. Therefore, medical records and death records in patients who have undergone surgical treatment for HNC were reviewed.

### 2.4. Statistical Analysis

We used numbers (proportions) and median and interquartile range (IQR) to represent categorical and continuous variables. We compared categorical variables by using the chi-square test, and we compared continuous variables with the Mann-Whitney U test. To balance the confounding factors in the PNI group, we calculated a propensity score for the PNI likelihood of each patient, using the patient’s demographic and characteristic covariates in a non-parsimonious multivariate logistic regression model. We then performed a 1:1 propensity score-matched analysis using the nearest-neighbor method, and a caliper of 0.1 SD units was used to construct matched pairs.

A boxplot analysis provided a data visualization method to present the median preoperative PNI by various complications. The logistic regression model was used to assess the association of the PNI group with overall and various complications. To improve the reliability of the results, two different adjustment models were applied before and after the propensity score matching dataset. Adjusted odds ratios (aOR) in the multivariate logistic regression model were calculated using the before propensity-score matching dataset (*n* = 2566), with confounders including variables with *p*-values < 0.05 in univariate models. In addition, propensity score-matched adjusted odds ratios (PSMs) were calculated using the propensity score-matched dataset (*n* = 1282), controlling for PNI and preoperative Hb. To improve the robustness of our results, two sensitivity analyses were performed. First, patients who undergo pre-operative adjuvant chemotherapy or immunotherapy may affect the calculation of PNI scores. Therefore, we excluded patients who received preoperative chemotherapy or immunotherapy, and a total of 1046 patients were analyzed in the propensity score matched dataset. Second, we used 40 points as the PNI cutoff. We constructed the crude and multivariate Cox’s proportional hazard models to estimate the recurrent rate and mortality rates during the follow-up period, and we used the Kaplan–Meier curves to express the estimated overall survival and recurrent rate. We used the log-rank tests to compare the differences between different groups.

We used SAS and R software (version 4.1.0; The Comprehensive R Archive Network: http://cran.rproject.org. accessed on 18 May 2021.) for all descriptions, statistical analyses, and visualization plots. We defined *p*-values less than 0.05 to be statistically significant in this study.

## 3. Results

The demographics and clinical characteristics of the patients are shown in Table 1. Patient age, education, cancer stage, primary site of cancer, ECOG score, operating time, pre-operative WBC count, pre-operative Hb level, pre-operative RDW, pre-operative absolute lymphocyte count, pre-operative neutrophil proportion, pre-operative albumin level, pre-operative ALT level, comorbidity disease with chronic kidney disease, receiving adjuvant chemotherapy, receiving adjuvant radiotherapy, receiving nutritional supplement, and receiving albumin supplement all showed statistically significant differences between the low (≤45) and high PNI (>45) group.

Among the 2566 patients, 925 had low PNI and 1641 had high PNI. After we performed a propensity score matching analysis, it generated 641 matched pairs, which were later divided into a low PNI group (*n* = 641) and high PNI group (*n* = 641) (Figure 1). All covariates were well-balanced. Preoperative Hb level, pre-operative absolute lymphocyte count, pre-operative albumin level, and pre-operative PNI had statistically significant differences between the low PNI group and high PNI group (Table 1).

### 3.1. Post-Operative Complications

A univariate logistic regression analysis demonstrated that the patients in the high PNI group had significant lower incidence of overall complications, medical complications, and pulmonary complications compared with the patients in the low PNI group (Table 2). The odds ratio in high PNI group of overall complications was 0.68 (95% confidence interval [CI] 0.56–0.84; *p*-value < 0.001), medical complications was 0.59 (95% CI 0.45–0.77; *p*-value < 0.001), pulmonary complications was 0.57 (95% CI 0.43–0.76; *p*-value < 0.001), and surgical complications was 0.82 (95% CI 0.64–1.05; *p*-value = 0.12).

**Figure 2:**
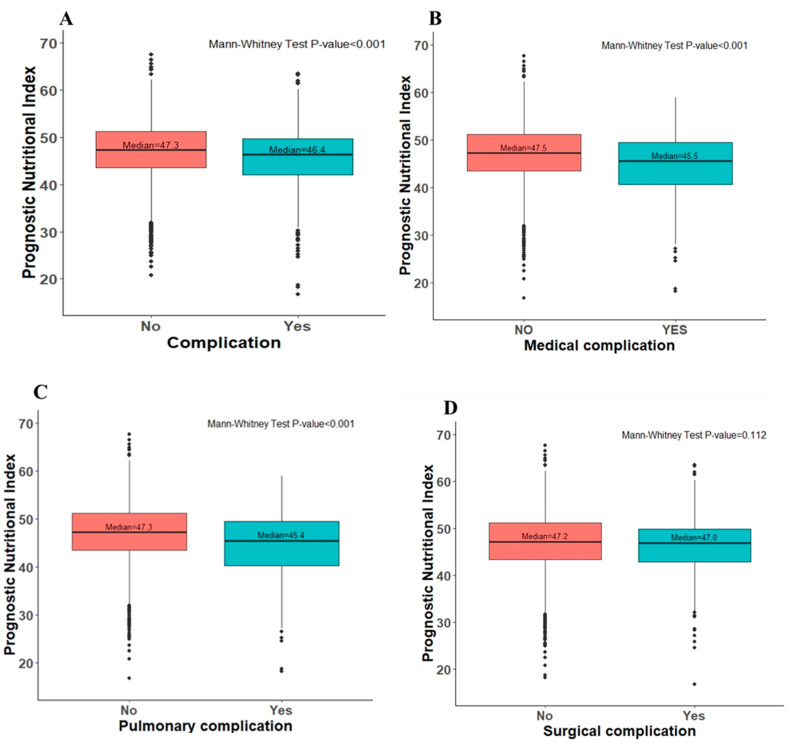
Box-plot analysis in the preoperative PNI score stratified by (**A**) overall complication, (**B**) medical complication, (**C**) pulmonary complication and (**D**) surgical complication event within 30 days after receiving resection surgery.

Similar results were also found in multivariate logistic regression and after propensity-score matching analysis (Table 2). In multivariate logistic regression, the odds ratio of overall complication in the high PNI group was 0.71 (95% CI 0.59–0.78; *p*-value < 0.001), medical complication was 0.70 (95% CI 0.54–0.81; *p*-value = 0.011), pulmonary complication was 0.71 (95% CI 0.53–0.82; *p*-value = 0.017), and surgical complication was 0.77 (95% CI 0.62–0.86; *p*-value = 0.022), compared to the low PNI group. The odds ratio of overall complications after propensity-score matching analysis was 0.72 (95% CI 0.54–0.96; *p*-value = 0.027), medical complication was 0.56 (95% CI 0.37–0.85; *p*-value = 0.007), pulmonary complication was 0.54 (95% CI 0.35–0.84; *p*-value = 0.007), and surgical complication was 0.92 (95% CI 0.66–1.28; *p*-value = 0.61) (Table 2). Although the propensity score match analysis was shown to be statistically non-significant in the incidence of surgical complications, a lower tendency could still be noted in the high PNI group. Preoperative PNI values were found to be lower in the postoperative complications group based on a box-plot analysis (Figure 2). 

In Supplementary Table S1, we present the sensitivity analysis and the results are consistent with the main findings of our research.

### 3.2. Risk Factor for Postoperative Complications

**Figure 3:**
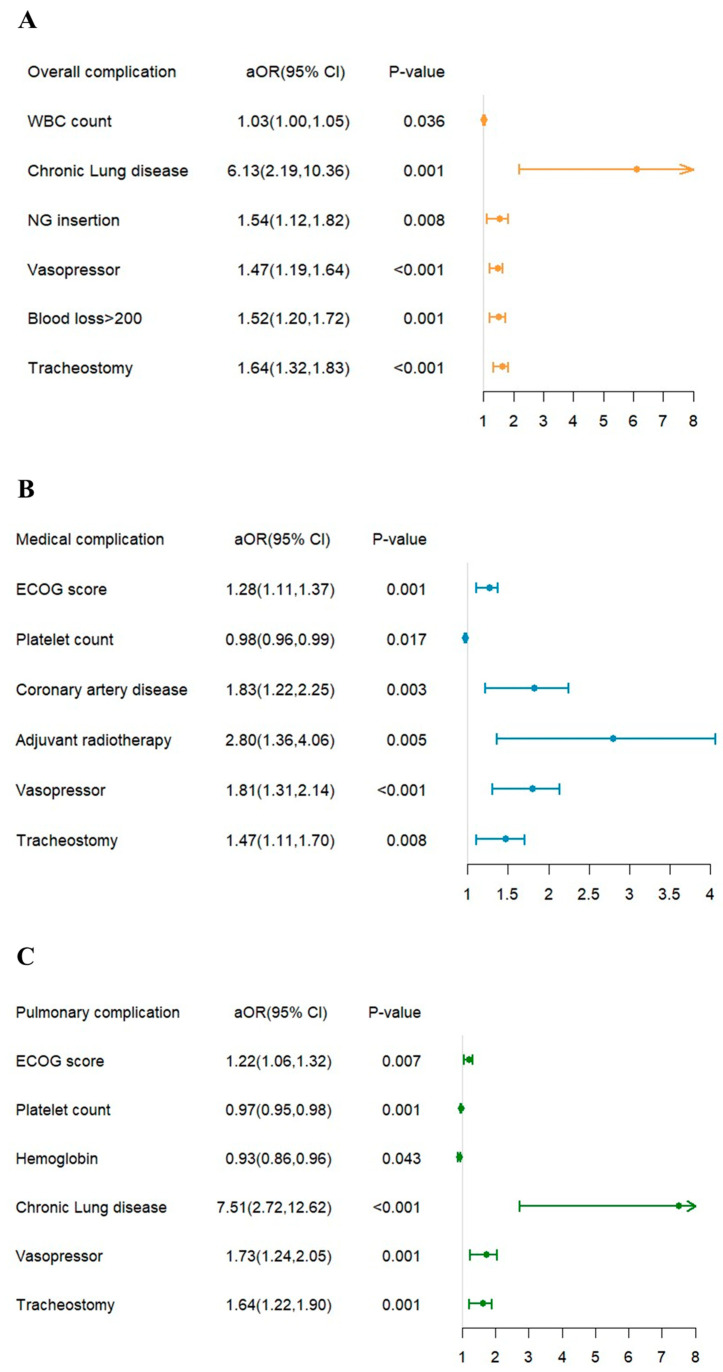

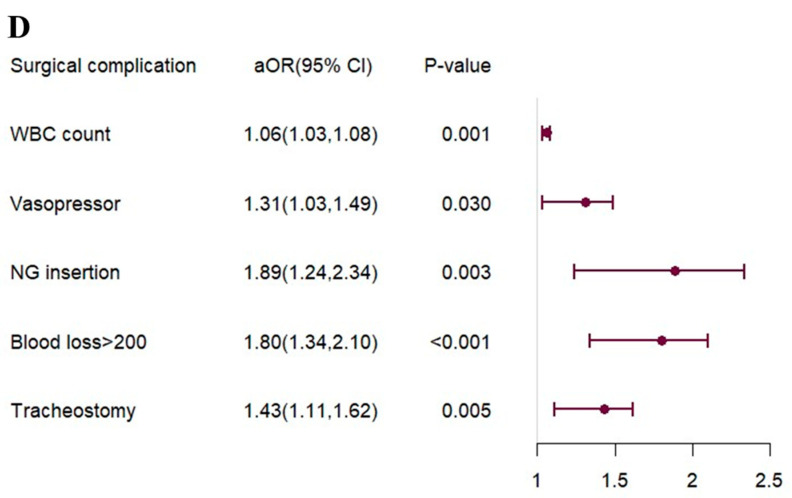
The significant risk factor for (**A**) overall complication, (**B**) medical complication, (**C**) pulmonary complication, and (**D**) surgical complication.

In risk factor analysis (Figure 3), WBC count (odds ratio [OR] = 1.03, [95% CI] = 1.00–1.05; *p*-value = 0.036), comorbidity with chronic lung disease (OR = 6.13, [95% CI] = 2.19–10.36; *p*-value = 0.001), and intra-operative NG insertion (OR = 1.54, [95% CI] = 1.12–1.82; *p*-value = 0.008), the patients who ever received vasopressors (OR = 1.47, [95% CI] = 1.19–1.64; *p*-value < 0.001), the patients who had the amount of blood loss >200 c.c during surgery (OR = 1.52, [95% CI] = 1.20–1.72; *p*-value = 0.001), and the patients who received tracheostomy during surgery (OR = 1.64, [95% CI] = 1.32–1.83; *p*-value < 0.001) were shown to have a higher risk for overall complications.

Higher ECOG score (OR = 1.28, [95% CI] = 1.11–1.37; *p*-value = 0.001), comorbidity with coronary artery disease (OR = 1.83, [95% CI] = 1.22–2.25; *p*-value = 0.003), the patients who received adjuvant radiotherapy (OR = 2.80, [95% CI] = 1.36–4.06; *p*-value = 0.005), the patients who ever received vasopressors (OR = 1.81, [95% CI] = 1.31–2.14; *p*-value < 0.001) and the patients who received tracheostomy during surgery (OR = 1.47, [95% CI] = 1.11–1.70; *p*-value = 0.008) were shown to have a higher risk for medical complications. Higher pre-operative PLT levels (OR = 0.98, [95% CI] = 0.96–0.99; *p*-value = 0.017) had a lower risk of medical complications.

Higher ECOG scores (OR = 1.22, [95% CI] = 1.06–1.32; *p*-value = 0.007), comorbidity with chronic lung disease (OR = 7.51, [95% CI] = 2.72–12.62; *p*-value < 0.001), patients who ever received vasopressors (OR = 1.73, [95% CI] = 1.24–2.05; *p*-value = 0.001) and the patients who received tracheostomy during surgery (OR = 1.64, [95% CI] = 1.22–1.90; *p*-value = 0.001) were shown to have higher risk for pulmonary complications. Higher pre-operative Hb levels (OR = 0.93, [95% CI] = 0.86–0.96; *p*-value = 0.043) and PLT levels (OR = 0.97, [95% CI] = 0.95–0.98; *p*-value = 0.001) were shown to have a lower risk of pulmonary complications.

Higher WBC counts (OR = 1.06, [95% CI] = 1.03–1.08; *p*-value = 0.001), patients who received vasopressors (OR = 1.31, [95% CI] = 1.03–1.49; *p*-value = 0.030), patients who received intra-operative NG insertion (OR = 1.89, [95% CI] = 1.24–2.34; *p*-value = 0.003), the patients who had the amount of blood loss >200 c.c during surgery (OR =1.80, [95% CI] = 1.34–2.10; *p*-value < 0.001) and the patients who received tracheostomy during surgery (OR = 1.43, [95% CI] = 1.11–1.62; *p*-value = 0.005) were shown to have higher risk for surgical complication.

### 3.3. Long-Term Outcome

**Figure 4:**
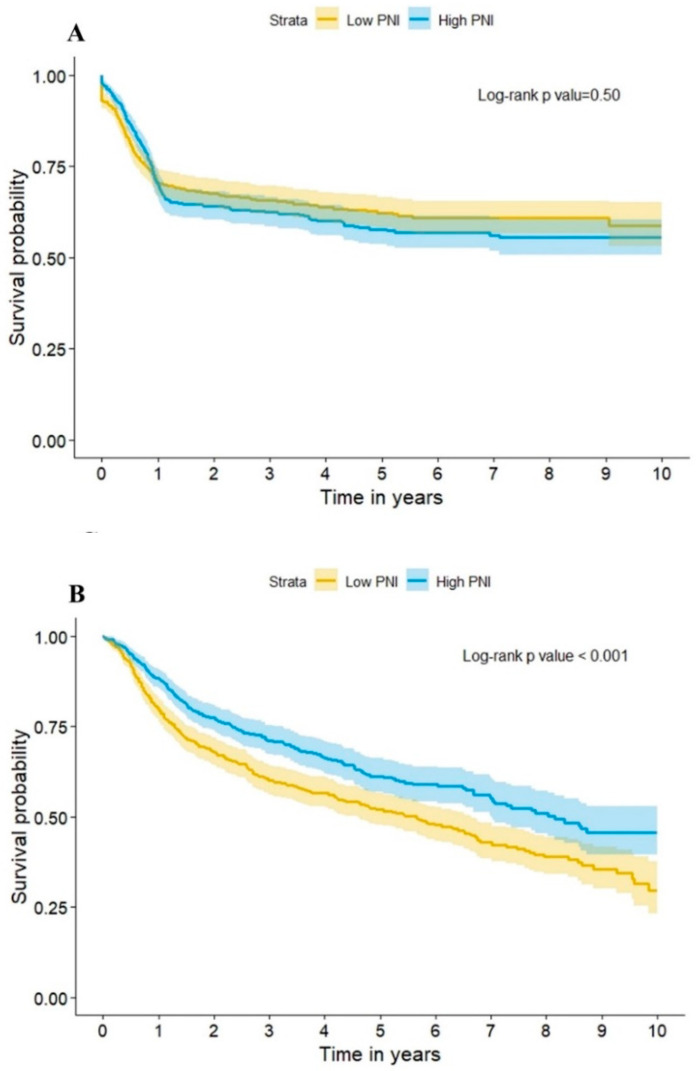

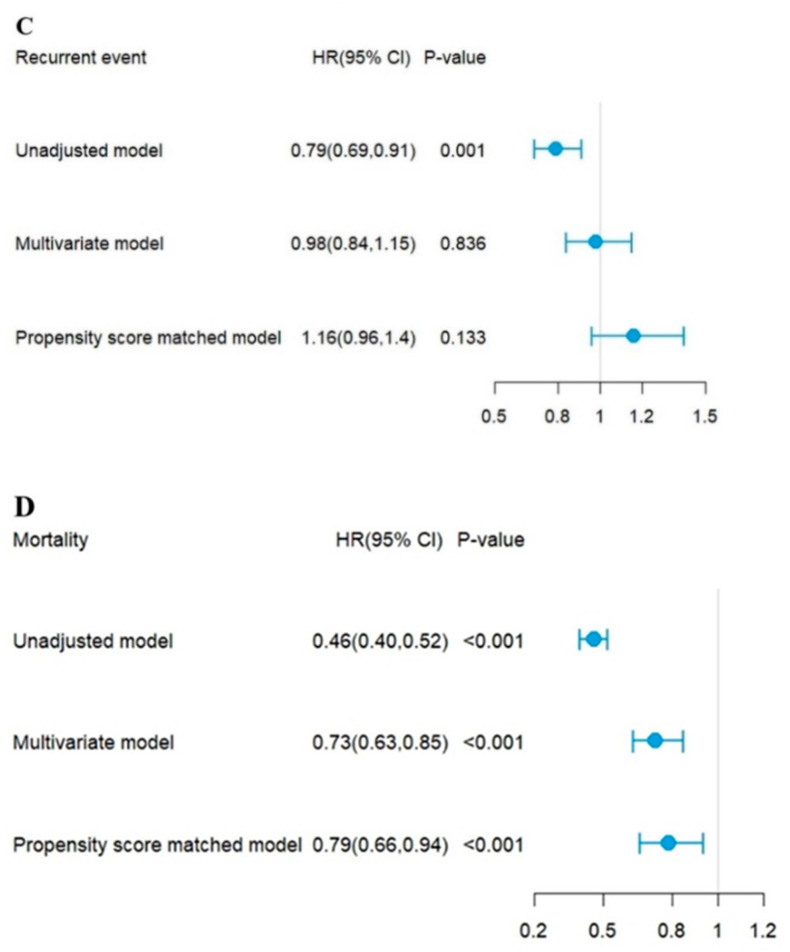
The impact of the PNI group on the long-term outcome using (**A**) Kaplan-Meier curves for the occurrence of cancer recurrence, (**B**) Kaplan-Meier curves for mortality, (**C**) Hazard ratio for the occurrence of cancer recurrence in the PNI group, and (**D**) Hazard ratio for mortality in the PNI group.

In the results of a Kaplan-Meier analysis, lower PNI was found to have lower overall survival compared with high PNI (*p* < 0.001) (Figure 4B). The Cox’s proportional hazard models showed that the high PNI group had lower mortality risk in the unadjusted model (hazard ratio = 0.46, [95% CI] = 0.40–0.52, *p*-value < 0.001), multivariate model (hazard ratio = 0.73, [95% CI] = 0.63–0.85, *p*-value < 0.001), and propensity score matching model (hazard ratio = 0.79, [95% CI] = 0.66–0.94, *p*-value < 0.001) (Figure 4D). The overall recurrence rate between the high and low PNI group was shown to be non-significant, but fewer recurrent events were noted within the first year of follow-up (Figure 4A). Higher PNI was significantly correlated with lower overall recurrent rate in an unadjusted model (hazard ratio = 0.79, [95% CI] = 0.69–0.91, *p*-value = 0.001), but was non-significant in a multivariate model (hazard ratio = 0.98, [95% CI] = 0.84–1.15, *p*-value = 0.836) and a propensity score matching model (hazard ratio = 1.16, [95% CI] = 0.96–1.4, *p*-value = 0.133) (Figure 4C).

## 4. Discussion

This study aimed to examine the relationship between preoperative PNI score and post-operative clinical outcomes in patients with HNC. To the best of our knowledge, this is the first study that compared both the short-term and long-term clinical outcomes between high and low PNI groups with more than 2000 HNC patients receiving a resection operation. There may be a great difference in patients’ features between high and low PNI groups, and confounding factors associated with patients’ underlying diseases may have also had an impact on the occurrence of the postoperative complications. Such conditions happen even if we adjusted the data by multivariate models; thus, a propensity score matching analysis was used in this study to correct for the influence of confounding factors and to reduce the possible bias when comparing the postoperative complication rate in patients with different PNI score. We believed that the final statistical results would reveal the difference caused by the different PNI score. The PNI score, in the literature review, was not only used as a biomarker that represents the nutritional status of the patient, but was also used as a prognostic indicator in patients with acute myocardial infarction, stroke, chronic obstructive lung disease, different types of cancers, and patients receiving different types of cancer-related surgeries [12,13,14,15,16,17,18,19,20,21]. In previously published data, the PNI score was used to assess the nutritional status and prognosis in HNC patients [22,23,24,25,26,27,28]. One meta-analysis showed that a lower level of pretreatment PNI score in HNC patients had a correlation with a worse survival rate [29]. Furthermore, the PNI score was found to have a correlation with treatment tolerance and toxicities in HNC patients receiving CCRT [30]. When it comes to the relationship between nutritional status and surgical outcomes of head and neck surgery, one study reported that malnutrition would give rise to a higher incidence of postoperative major complications after major surgery for advanced HNC, and the loss of more than 10% of body weight 6 months before surgery was the most prominent parameter [31]. Takayuki Imai found that the occurrence rate of postoperative complications was affected by the PNI score in patients who received head and neck surgery with free flap reconstruction [32].

In this study, we used the preoperative PNI score as the biomarker to predict postoperative complications. Lyell used the preoperative serum albumin level as a biomarker to predict survival prognosis and the occurrence of any postoperative complications in patients receiving pelvic exenteration surgery in colorectal, genitourinary, and gynecologic cancer [33]. Cheong Wei Eu found that a decrease in serum albumin levels (<35 g/L) results in an increased rate of postoperative surgical site infection [34]. Caburet reported Nutrition Risk Index (NRI, NRI = 1.519 × serum albumin (g/L) + 41.7 × (present weight/usual weight)) to be an independent risk factor for postoperative infection-related and healing-related complications in HNC patients receiving surgery [35]. Another study showed that preoperative CONUT score (calculated by serum albumin level, serum total cholesterol level, and serum lymphocyte counts) predicts the incidence of postoperative complications in hip fracture patients and patients with pancreatic cancer receiving pancreaticoduodenectomy (PD) [36,37]. Our study showed that a low PNI score was independently associated with a higher risk for postoperative overall complications, medical complications, and pulmonary complications, followed by the surgical resection of HNC. The results were in agreement with previous literature that the preoperative nutrition status may have a strong correlation with postoperative major and overall complications. Under the same propensity score, the relationship between PNI score and the incidence of surgical complications in our study proved to be non-significant; however, a trend toward lower surgical complications in the high PNI group could still be observed. The reason why the results did not reach significance might be that the cutoff we set to distinguish between the low and high PNI groups needs to be adjusted or the sample size needs to be larger, which may require further research to clarify. Another possible reason for this non-significant result might be because of the definition of surgical complication set in our study being complications that occurred within 30 days after surgery and requiring re-operation. Wound dehiscence, wound infection, poor wound healing, or postoperative wound bleeding were the most common postoperative complications that happened in our daily practice requiring additional surgery. Nevertheless, initial treatment with re-operation was not the only treatment modality for such conditions. Antibiotic treatment or proper wound dressing may serve as another effective treatment for wound dehiscence, wound infection, or poor wound healing. Conservative treatment for postoperative wound bleeding may also be used in clinical practice. In other words, re-operation may not always be chosen as the treatment modality in the first 30 days, which may lead to the non-significant result in the incidence of surgical complications in our study.

We set 45 as the cut-off value of PNI in our study. Onodera published the first study that showed that the low status of the preoperative PNI score (PNI ≤ 40) had a higher complication rate for gastric or colorectal cancer patients receiving anastomosis [11]. Another study showed that a cutoff value of PNI ≤ 40 had a predictive value of postoperative complications in HNC surgery with free flap reconstruction [32]. Guller reported low PNI (≤45) to be associated with worse overall survival and progression-free survival in advanced HNC patients receiving immunotherapy [8]. Which cut-off value should be set was still a controversial issue.

In addition to PNI score, we assumed that other factors related to nutrition status, such as hemogram and postoperative feeding methods, might also be associated with the occurrence of postoperative complications. The results in our study showed consistency with this hypothesis. Michael K Ghiam reported that dysphagia accounts for up to 15% of all 30-day readmission of patients after head and neck surgery [38]. Patients with intraoperative nasogastric (NG) tube insertion, compared with patients with early oral feeding, are at a higher risk of overall complications, possibly due to a large defect in the upper aerodigestive tract or expected impaired swallowing function following cancer resection, such as free flap reconstruction, which is also associated with a more complex postoperative condition that may require more time to restore the swallowing function. In addition, more complicated postoperative conditions might lead to higher rates of re-operation, which might explain the higher incidence of surgical complications. When it comes to the surgical routine in our hospital, patients receiving hemi-glossectomy (or a larger resection size) would receive temporary NG tube insertion. An NG tube will also be inserted to postoperative patients with T2 or more advanced cancers which originated from other sites of the oral cavity region (such as the buccal area or gingiva, whether the local flap was performed to cover the excisional defect or not) or other head and neck regions (such as oropharynx and hypopharynx). Furthermore, patients that receive tracheostomy or free flap re-construction surgery will also receive NG tube intake after surgery in our hospital. To sum up, an NG tube will be inserted when a patient receives HNC resection surgery for (1) more advanced cancer, (2) cancer that involves an overlapping anatomical region, or (3) a patient who receives any other procedure (such as tracheostomy) that postoperative dysphagia was highly suspected.

One meta-analysis reported that hemodynamic therapy, which included perioperative fluid, inotrope, or vasopressor administration, affects the occurrence of postoperative pulmonary complications [39]. The patients receiving vasopressors within 30 days following an operation indicated an unstable hemodynamic status, which might explain the higher incidence of overall complications, medical complications, pulmonary complications, and surgical complications in our study.

Higher preoperative Hb levels and PLT levels were correlated with the lower incidence of postoperative pulmonary complications. One meta-analysis reported preoperative anemia to be associated with poor outcomes after surgery [40]. Evie Yeap reported that preoperative thrombocytopenia and anemia were associated with the occurrence of postoperative anastomotic leak and venous thromboembolism, as well as the higher rate of 30-day mortality in patients receiving colorectal resection [41]. The results of our study showed consistency with the previous literature.

### Limitations and Future Research

The first limitation of the study was that it was a single institution study. However, with reference to the official annual report of the cancer registry in Taiwan in 2019 [42], the number of new diagnoses in Changhua Christian Hospital and the number of new diagnoses in all of Taiwan were 476 and 5340 in oral cancer, 114 and 1649 in oropharyngeal cancer, 97 and 1215 in hypopharyngeal cancer, as well as 56 and 846 in laryngeal cancer, respectively. The number of patients in Changhua Christian Hospital accounts for approximately 6–8% of all patients in Taiwan, which means that our findings may still be representative. The second limitation was that our study design was a retrospective cohort study, making it relatively difficult to confirm causal relationships between variables. Although we used a propensity score matching analysis to adjust some possible confounding factors, further prospective studies may be needed.

## 5. Conclusions

The PNI score is a significant independent predictor of both short-term postoperative complications and long-term survival in HNC patients undergoing surgical resection. We recommend the preoperative testing and evaluation of HAC patients to identify low PNI and high-risk groups for postoperative surveillance. Further intervention studies are still needed to determine whether nutritional advice and interventions for low PNI or high-risk patients are needed to reduce postoperative complications.

## Figures and Tables

**Table 1:** The demographics and clinical characteristics divided by cut-off value of PNI score = 45 before and after propensity score matching.

	Before Propensity Score Matching			After Propensity Score Matching		
	Low PNI (*n* = 925)	High PNI (*n* = 1641)	*p*-Value	Low PNI (*n* = 641)	High PNI (*n* = 641)	*p*-Value
**BMI (kg/m^2^)**	22.7 (20.3, 25.6)	24.6 (22.2, 27.2)	<0.001	23.3 (20.8, 26.6)	23.6 (21.3, 26.1)	0.303
**Gender, Male**	886 (95.8%)	1557 (94.9%)	0.304	612 (95.5%)	611 (95.3%)	0.894
**Age (years)**	60 (53, 67)	56 (49, 64)	<0.001	59 (52, 67)	59 (52, 68)	0.603
**Education**			<0.001			0.755
Primary school or below	420 (45.4%)	554 (33.8%)		273 (42.6%)	272 (42.4%)	
Secondary school	270 (29.2%)	548 (33.4%)		193 (30.1%)	189 (29.5%)	
High school	198 (21.4%)	438 (26.7%)		149 (23.2%)	152 (23.7%)	
University or above	37 (4.0%)	101 (6.1%)		26 (4.1%)	28 (4.3%)	
**Stage**			<0.001			0.316
I	183 (19.8%)	529 (32.3%)		155 (24.2%)	142 (22.2%)	
II	145 (15.7%)	366 (22.3%)		108 (16.8%)	123 (19.2%)	
III	122 (13.2%)	215 (13.1%)		79 (12.3%)	96 (15%)	
IV	475 (51.4%)	531 (32.4%)		299 (46.6%)	280 (43.7%)	
**Lymphatic invasion**	293 (31.7%)	431 (26.3%)	0.003	190 (29.6%)	215 (33.5%)	0.133
**Tumor Grade**			0.744			0.916
Well-differentiated	88 (9.5%)	178 (10.8%)		64 (10%)	63 (9.8%)	
Moderately differentiated	671 (72.5%)	1181 (72%)		462 (72.1%)	456 (71.1%)	
Poorly differentiated	66 (7.1%)	112 (6.8%)		49 (7.6%)	48 (7.5%)	
Undifferentiated/unknown	100 (10.8%)	170 (10.4%)		66 (10.3%)	74 (11.5%)	
**Tumor site**			<0.001			0.233
Oral cavity	656 (70.9%)	1346 (82%)		477 (74.4%)	500 (78%)	
Oropharyngeal	120 (13%)	154 (9.4%)		73 (11.4%)	74 (11.5%)	
Hypopharyngeal	95 (10.3%)	84 (5.1%)		52 (8.1%)	44 (6.9%)	
Laryngeal cancer	47 (5.1%)	54 (3.3%)		34 (5.3%)	21 (3.3%)	
Nasal cavity and sinus	7 (0.8%)	3 (0.2%)		5 (0.8%)	2 (0.3%)	
**Smoking habits**			0.008			0.310
Never	64 (6.9%)	116 (7.1%)		52 (8.1%)	44 (6.9%)	
Quit	563 (60.9%)	1090 (66.4%)		383 (59.8%)	409 (63.8%)	
Current	298 (32.2%)	435 (26.5%)		206 (32.1%)	188 (29.3%)	
**Betel nut habits**			0.071			0.976
Never	60 (6.5%)	117 (7.1%)		47 (7.3%)	49 (7.6%)	
Quit	756 (81.7%)	1281 (78.1%)		516 (80.5%)	515 (80.3%)	
Current	109 (11.8%)	243 (14.8%)		78 (12.2%)	77 (12%)	
**Alcohol habits**			0.732			0.972
Never	471 (50.9%)	831 (50.6%)		325 (50.7%)	329 (51.3%)	
Quit	124 (13.4%)	205 (12.5%)		83 (12.9%)	81 (12.6%)	
Current	330 (35.7%)	605 (36.9%)		233 (36.3%)	231 (36%)	
**ECOG**			0.001			0.796
0	822 (88.9%)	1529 (93.2%)		582 (90.8%)	582 (90.8%)	
1	50 (5.4%)	67 (4.1%)		31 (4.8%)	37 (5.8%)	
2	10 (1.1%)	17 (1%)		8 (1.2%)	8 (1.2%)	
3	3 (0.3%)	6 (0.4%)		2 (0.3%)	1 (0.2%)	
4	40 (4.3%)	22 (1.3%)		18 (2.8%)	13 (2%)	
Operating time, min	424 (285, 565)	375 (261, 525)	<0.001	421 (281, 560)	400 (269, 540)	0.298
Blood loss, cc	300 (150, 400)	200 (100, 300)	<0.001	250 (150, 400)	200 (150, 350)	0.129
Tracheostomy	635 (62.0%)	953 (53.1%)	<0.001	435 (60.5%)	442 (60.63%)	0.960
**Preoperative laboratory tests**						
Hemoglobin (g/dL)	12.6 (11.1, 14)	14.4 (13.3, 15.3)	<0.001	13.2 (11.8, 14.3)	14 (12.9, 15)	<0.001
Platelet count (1000/μL)	220 (168, 283)	222 (183, 267)	0.313	224 (173, 281)	222 (183, 272)	0.855
RDW (%)	14.1 (13.3, 15.4)	13.5 (13, 14.3)	<0.001	13.8 (13.2, 14.8)	13.8 (13.2, 14.8)	0.707
WBC count (1000/μL)	6.5 (5.1, 8.5)	7.3 (6.1, 8.7)	<0.001	6.6 (5.2, 8.8)	7 (5.9, 8.5)	0.008
Absolution lymphocyte count (1000/μL)	1.2 (0.8, 1.5)	1.8 (1.5, 2.2)	<0.001	1.3 (1, 1.6)	1.6 (1.3, 1.9)	<0.001
Neutrophil (%)	69.6 (62.4, 75.9)	63.3 (57.2, 69.1)	<0.001	67 (60.5, 73.8)	66.8 (61.5, 71.7)	0.366
Albumin (g/dL)	3.5 (3.2, 3.7)	4.1 (3.9, 4.3)	<0.001	3.5 (3.3, 3.7)	4.1 (3.9, 4.3)	<0.001
Creatinine (mg/dL)	0.9 (0.7, 1.1)	0.9 (0.8, 1)	0.811	0.9 (0.8, 1.1)	0.9 (0.8, 1.1)	0.625
ALT (U/L)	19 (15, 29)	24 (17, 34)	<0.001	20 (15, 29)	20 (16, 31)	0.067
PNI score	41.7 (38.1, 43.6)	49.9 (47.4, 52.8)	<0.001	42.2 (39.7, 43.9)	48.7 (46.7, 51)	<0.001
**Comorbidity disease**						
Hypertension	330 (35.7%)	565 (34.4%)	0.525	229 (35.7%)	223 (34.8%)	0.726
Diabetes mellitus	208 (22.5%)	314 (19.1%)	0.043	139 (21.7%)	138 (21.5%)	0.946
Coronary artery disease	85 (9.2%)	125 (7.6%)	0.163	60 (9.4%)	56 (8.7%)	0.697
Chronic Kidney Disease	31 (3.4%)	17 (1%)	<0.001	13 (2%)	15 (2.3%)	0.702
Chronic Lung disease	10 (1.1%)	6 (0.4%)	0.027	5 (0.8%)	6 (0.9%)	0.762
**Treatment**						
Adjuvant chemotherapy	248 (26.8%)	218 (13.3%)	<0.001	114 (17.8%)	122 (19%)	0.564
Adjuvant radiotherapy	34 (3.7%)	4 (0.2%)	<0.001	4 (0.6%)	4 (0.6%)	1.000
Vasopressor	641 (69.3%)	1091 (66.5%)	0.144	434 (67.7%)	421 (65.7%)	0.441
Nutritional supplement	234 (25.3%)	171 (10.4%)	<0.001	114 (17.8%)	113 (17.6%)	0.942
Glutamine supplement	9 (1%)	3 (0.2%)	0.005	4 (0.6%)	3 (0.5%)	0.705
Amino acid supplement	9 (1%)	9 (0.5%)	0.216	5 (0.8%)	6 (0.9%)	0.762
Albumin supplement	223 (24.1%)	163 (9.9%)	<0.001	109 (17%)	108 (16.8%)	0.941
**Feeding method**						
Jejunostomy	13 (1.4%)	10 (0.6%)	0.040	5 (0.8%)	6 (0.9%)	0.762
NG insertion	757 (81.8%)	1423 (86.7%)	0.001	546 (85.2%)	549 (85.6%)	0.812
Propensity Score	0.5 ± 0.2	0.7 ± 0.2	<0.001	0.6 ± 0.2	0.6 ± 0.2	0.981
**30 days short-term outcome**						
Complication	208 (22.5%)	272 (16.6%)	<0.001	139 (21.7%)	108 (16.8%)	0.028
Medical complication:	108 (11.7%)	118 (7.2%)	<0.001	68 (10.6%)	39 (6.1%)	0.003
Pulmonary complication	100 (10.8%)	106 (6.5%)	<0.001	62 (9.7%)	35 (5.5%)	0.004
Surgical complication:	115 (12.4%)	171 (10.4%)	0.120	81 (12.6%)	75 (11.7%)	0.608
**Long-term outcome**						
Recurrent	325 (36.3%)	521 (33.4%)	0.155	214 (34.6%)	235 (38.7%)	0.138
Death	494 (55.1%)	490 (31.5%)	<0.001	307 (49.6%)	212 (34.9%)	<0.001

**Table 2:** Univariate and multivariate analyses of postoperative complication events in the PNI group using before and after propensity score-matched datasets.

	Univariate Analysis		Multivariate Analysis		Propensity Score Match Analysis	
	cOR (95% CI)	*p*-Value	aOR (95% CI)	*p*-Value	PSM (95% CI)	*p*-Value
Overall Complication						
Low PNI	1		1		1	
High PNI	0.68 (0.56, 0.84)	<0.001	0.71 (0.59, 0.78)	<0.001	0.72 (0.54, 0.96)	0.027
Medical complication						
Low PNI	1		1		1	
High PNI	0.59 (0.45, 0.77)	<0.001	0.70 (0.54, 0.81)	0.011	0.56 (0.37, 0.85)	0.007
Pulmonary complication						
Low PNI	1		1		1	
High PNI	0.57 (0.43, 0.76)	<0.001	0.71 (0.53, 0.82)	0.017	0.54 (0.35, 0.84)	0.007
Surgical complication						
Low PNI	1		1		1	
High PNI	0.82 (0.64, 1.05)	0.120	0.77 (0.62, 0.86)	0.022	0.92 (0.66, 1.28)	0.610

(OR, odds ratio; CI, confidence interval; cOR, crude odds ratio; aOR, the multivariate-adjusted odds ratio; PSM, the odds ratio using propensity score matching data.) cOR was estimated using a logistic regression model, a model including PNI only. aOR was estimated using a logistic regression model with stepwise elimination procedure, confounders including the variables with a *p*-value < 0.05 in a univariate model were included in a multivariate model. PSM was estimated using propensity score matching data and adjusted for PNI and preoperative Hb, while the propensity score was performed by using the variables shown in Table 1.

## Data Availability

Not applicable.

## Supplementary Materials

The following supporting information can be downloaded at: https://www.mdpi.com/article/10.3390/nu14245331/s1, Table S1: Sensitivity Analysis of Excluding Patients who Received Preoperative Chemotherapy or Immunotherapy and Define PNI > 40 as the High PNI group.
