# Supplementary material for: Relationship between Preoperative Nutritional Status and Clinical Outcomes in Patients with Head and Neck Cancer
Source: Nutrients. 2022 Dec 15;14(24):5331. doi: 10.3390/nu14245331 (PMC9782741; doi:10.3390/nu14245331)
Supplement: Supplementary file 1 [file nutrients-14-05331-s001.zip › nutrients-2056685-supplementary.pdf]

Table S1. Sensitivity Analysis of Excluding Patients who Received Preoperative Chemotherapy or Immunotherapy and Define PNI > 40 as the High PNI group.

	**Propensity score match analysis**	
	**PSM (95% CI)**	***p*-Value**
Excluding patients who received preoperative chemotherapy or immunotherapy (*n* = 1046)		
Overall Complication		
High PNI vs. Low PNI	0.67(0.49,0.79)	0.014
Medical complication		
High PNI vs. Low PNI	0.59(0.38,0.73)	0.017
Pulmonary complication		
High PNI vs. Low PNI	0.56(0.36,0.72)	0.016
Surgical complication		
High PNI vs. Low PNI	0.85(0.57,1.03)	0.400
Define PNI > 40 as the High PNI group (*n* = 1282)		
Overall Complication		
High PNI vs. Low PNI	0.64(0.43,0.78)	0.023
Medical complication		
High PNI vs. Low PNI	0.40(0.24,0.51)	<0.001
Pulmonary complication		
High PNI vs. Low PNI	0.40(0.24,0.52)	0.001
Surgical complication		
High PNI vs. Low PNI	0.92(0.57,1.18)	0.740
